# Modification, Implementation, and Evaluation of a Community-Based Dementia Information Session

**DOI:** 10.1093/geroni/igaf122.549

**Published:** 2025-12-31

**Authors:** Teresa McCarthy

**Affiliations:** University of Minnesota, Minneapolis, Minnesota, United States

## Abstract

Building empathy and understanding for those with cognitive decline is a fundamental component of Dementia Friendly communities. The Dementia Friends Information Session is one hour of content delivered by community volunteers to community members in a “train the trainer” model. The intent of the sessions is to provide face-to-face information about “what dementia is and how it affects people, and that each individual can make a difference for people touched by dementia.” The content was developed in the UK and licensed in Minnesota to our Area Agency on Aging. In 2019, the University of Minnesota HRSA-funded Geriatric Work Force Enhancement Program (GWEP) successfully implemented this model to educate health science students at the University of Minnesota. When the COVID 19 pandemic precluded these in-person sessions, the University transitioned the content to on-line virtual sessions. University relations with the community owners of the content allowed the program to be quickly modified and delivered virtually across the state despite the pandemic. This paper reviews the transition of this well-established community-based program to virtual delivery due to the COVID pandemic. The outcome of the intervention was assessed for a population of health sciences students utilizing the Dementia Attitude Scale. A well-established community-based information session was adapted for academic education. The academic relationship facilitated a rapid transformation of the face-to-face sessions to virtual delivery in the community that sustained the program through the COVID pandemic.

